# Characterization of the gut microbiome and resistome of piglets supplemented with low, moderate, and high concentrations of zinc oxide

**DOI:** 10.1128/spectrum.00059-26

**Published:** 2026-09-03

**Authors:** Alexandra Langlois, Antony T. Vincent, Karoline Lauzon, Jean-Simon Brouard, Danyel Bueno Dalto, Nathalie Gagnon, Guylaine Talbot, Jérôme Lapointe, Dominic Poulin-Laprade

**Affiliations:** 1 Sherbrooke Research and Development Centre, Agriculture and Agri-Food Canada, Sherbrooke, Quebec, Canada; 2 Animal Science Department, Faculty of Agriculture and Food Sciences, Université Laval 4440, Québec City, Quebec, Canada; The University of Tennessee Southern, Pulaski, Tennessee, USA; ChongQing Academy of Animal Sciences, Rongchang, Chongqing, China; University of Pretoria, Pretoria, South Africa

**Keywords:** zinc oxide, antimicrobial resistance, metagenomics, pig, post-weaning

## Abstract

The livestock sector is standing out for its substantial use of antimicrobials but is also investing in the development of mitigation strategies. Supranutritional concentrations of zinc (Zn) are served to piglets to prevent post-weaning diarrhea. This practice is detrimental to the piglet’s assimilation of nutrients and can impact the environment through the excretion of unassimilated Zn, contributing to the persistence of antibiotic resistance by co-selection. In this study, the ileum and colon microbiome of piglets fed low (175 ppm), moderate (953 ppm), or high (2,991 ppm) concentrations of zinc oxide for 21 days after weaning were investigated using metagenomics and 16S rRNA amplicon sequencing. The pH and volatile fatty acids (VFA) were measured in cecum and colon. The colon resistome was investigated using metagenomics and PCR. The high Zn supplementation led to an increase in pH and a decrease in the VFA in both the cecum and colon. The bacterial diversity in colon was negatively impacted by the high Zn diet, with thriving *Clostridiaceae* and depletion of beneficial populations (e.g., *Lactobacillaceae* and *Selenomonadaceae*). Of concern, higher abundances of *bla*_CTX-M_ and *intI1* were observed in piglets fed with the moderate Zn diet. The findings from this study support the limitation of Zn to low concentrations.

**IMPORTANCE:**

Developing alternatives to antibiotics is imperative to mitigate the global antimicrobial resistance crisis, particularly within the livestock sector, the predominant consumer of these agents. Yet, interventions introduced without due consideration may precipitate unintended consequences. In North American swine production, supranutritional zinc supplementation in pig feed has been adopted to prevent post-weaning diarrhea and sustain profitability. However, excessive zinc disrupts iron and copper metabolism and perturbs mitochondrial homeostasis, impairing energy metabolism, antioxidant defenses, and innate immunity. Moreover, high dietary zinc increases the excretion of this heavy metal, contributing to environmental pollution and fostering antibiotic-resistant bacteria through co-selection. This study provides further evidence supporting the restriction of zinc supplementation to nutritional needs, as supranutritional diets compromised the fermentative capacity of the pig gut microbiome and increased the frequency of clinically relevant antimicrobial resistance markers.

## INTRODUCTION

Weaning is a critical time in the life of growing pigs as piglets are separated from their mother, often transported to a new building, mixed with piglets from different litters in which a new hierarchy has to be established, and switch from a milk-oriented to a solid diet, all of this while their immune and digestive systems are still immature (1–3). Those stressful conditions can disrupt the intestinal morphology causing the so-called post-weaning diarrhea, which can lead to high mortality rates, animal welfare concerns, and significant economic losses (1). Therefore, antibiotics have been used prophylactically for decades to mitigate the negative consequences of early weaning (4). However, prophylactic antibiotics should be used carefully as it can select for antibiotic-resistant bacteria. As an alternative to the use of antibiotics, it is a common practice in commercial farming to supplement pig feed with high levels of zinc (Zn), often in the form of zinc oxide (ZnO) (5). The Guideline on Nutrient Requirements of the National Research Council (NRC) states that piglets weighing 5–7 kg, aged approximately 21 days, require 100 ppm of Zn (100 mg of Zn per kg of feed) to sustain a good health (6). However, levels of Zn reaching 3,000 ppm are commonly used as dietary supplement in commercial operations worldwide (7, 8), including in Canada (9). This practice poses environmental concerns as piglets do not fully assimilate high concentrations of Zn, leading to substantial Zn excretion, that accumulates in soil through repeated manure spreading (10, 11). In Canada, legislations regarding manure application are specific to each province and territory. Currently, this regulation does not cover the level of all minerals encountered in fertilizers, like copper (Cu) and Zn. Other than environmental issues, high dietary Zn level is also a concern in terms of co-selection for antimicrobial-resistant bacteria in niches harboring high concentrations of metals (12–14). As antibiotic and metal resistance genes (MRGs) can occur on the same genetic element, the presence of metals in a given environment, for instance in the pig gastrointestinal tract or soil, can exert a selection pressure for metal- as well as biocide- and antibiotic-resistant bacteria (15, 16).

Consequently, the European Union has legislated on the concentration of Zn in pig feed and banned the use of concentrations beyond 150 ppm of Zn (17, 18). There have also been discussions in Canada on the use of Zn in commercial swine feed (19), and current regulations set the Zn maximum nutrient value at 300 ppm for swine (except 3,000 ppm for starters) (20). Science-based evidence is required to inform policy, capture the complexity of the biological processes leading to antimicrobial resistance (AMR) expansion, and translate it into well-informed decisions and successful interventions. Many studies already focused on the impact of various concentrations of in-feed Zn on the health and robustness of piglets (21–26) or Zn excretion and environmental issues (27–31). Other studies also used shotgun metagenomics to evaluate the impact of dietary Zn on the gut microbiome or resistome of pigs (32–34), but our study was conducted in controlled conditions, limiting external factors for an optimal focus on the impacts of varying Zn concentrations on the gut microbiome and resistome. This study used sequencing and PCR methods to provide evidence regarding the impact of Zn concentrations on piglet gut microbiome and resistome and is complementary to previous work in which collaborators investigated, in the same animals, the impact of Zn in pig feed on the metabolism of Zn, Cu, and iron (35–37). For the current study, we hypothesized that contrasting dietary ZnO levels would modulate the gut microbiome and that piglets fed with higher concentrations of ZnO would carry more antimicrobial resistance genes (ARGs) and MRGs.

## MATERIALS AND METHODS

### Animals, treatments, and samples

**Fig 1:**
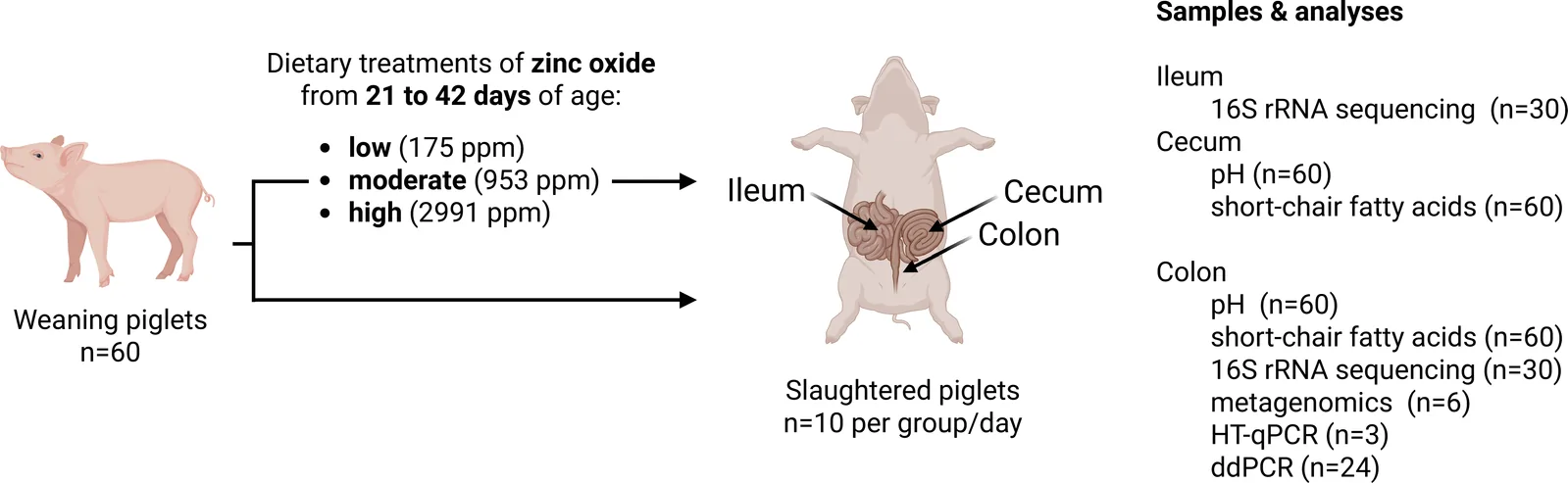
Schematic representation of the animals, treatments, samples, and analyses in this study. Created in BioRender. Langlois (2026) https://BioRender.com/bo88h9b.

The experimental procedures were approved by the Institutional Animal Care Committee of the Sherbrooke Research and Development Centre (protocol #555) according to the Canadian Council on Animal Care (38). Care of pigs was conducted according to the National Farm Animal Care Council guidelines (39). The experimental set up was described in detail by Dalto et al. (35). Briefly, 60 Yorkshire-Landrace × Duroc lactating piglets were selected at 14 days of age and were used in a generalized randomized complete block design with four blocks. At weaning, piglets aged 21 days were randomly assigned to one of the three experimental diets containing either low (175 ppm), moderate (953 ppm), or high (2,991 ppm) concentrations of ZnO (Fig. 1). The low concentration meets the nutritional requirements of pigs, while the moderate and high concentrations reflect standard practice in industry. The total Zn concentration in each diet was confirmed experimentally by SGS Canada Inc. using Inductively Coupled Plasma-Optical Emission Spectroscopy based on the AOAC Official Method 965.09 (40). Experimental diets were fed to piglets for a period of 3 weeks until day 42. Ten piglets per treatment were slaughtered at 21 days of age (D21), prior to diet administration, and at 42 days of age (D42).

Ileum, cecum, and colon digesta were sampled at slaughter. The pH was immediately measured directly into the cecum. Colon digesta (~5 g) was diluted 1:10 with tap water at pH 7, and then the pH was measured. Ileum digesta samples (~2 g) were aliquoted and snap-frozen in liquid nitrogen. Cecum and colon digesta were diluted in 0.9% saline (1:1), subsampled, and snap-frozen in liquid nitrogen. Samples intended for VFA analysis were kept at −20°C, whereas all the other samples were kept at −80°C until further analysis.

### Quantification of volatile fatty acids

Volatile fatty acids were quantified in cecal and colonic samples (Fig. 1) as fermentation mainly occurs at those sites. Briefly, samples were diluted 1:4 in bidistilled water, vortexed, and centrifuged at 12,000 × *g* for 15 min. A weight of 1.3 g of supernatant was homogenized with 250 µL of 0.5 M H_2_SO_4_ and centrifuged at 12,000 × *g* for 15 min. Then, 0.5 mL of the supernatant was combined 1:1 with the internal standard 2-ethylbutyric acid at a concentration of 2 g/L and mixed with 0.15 g of Dowex 50W-X8 resin beads (Millipore Sigma, QC, Canada). The mixture was vortexed and incubated 10 min at room temperature to let the beads settle before passing through a 0.45 µm Minisart filter (Sartorius, Germany). Analyses were performed using a Perkin-Elmer gas chromatograph, model Clarus 580 (Perkin-Elmer Corporation, Shelton, CT, USA) coupled to a high-resolution DB-FFAP column connected to a flame ionization detector. VFA concentrations, expressed in mg/L, were deduced from measures obtained for a standard solution containing acetic, propionic, butyric, isobutyric, valeric, isovaleric, and caproic acids at 2 g/L in reverse-osmosed deionized water with a resistivity >10 megaohm/cm.

### Shotgun metagenomics

Prior to DNA extractions, equal volumes of colon digesta collected during two blocks (out of four) at a given sampling day (D21 or D42) and Zn supplementation (low, moderate, or high) were pooled, leading to 12 pools of samples each representing five individual piglets.

DNA extractions were conducted as described by Zaheer et al. (41) with minor modifications. Frozen diluted colon digesta were thawed and diluted again with 0.9% saline (1:1), and then 350 µL was transferred to a screw-cap tubes containing 0.3 g of 0.1 mm Zirconia-Silicate beads and 0.1 g of 0.5 mm Zirconia-Silicate beads as well as 1 mL of resuspension buffer (600 mM NaCl, 120 mM Tris-HCl, 60 mM EDTA, 200 mM guanidine isothiocyanate) and 5 µL of β-mercaptoethanol. Then, 200 μL of 10% sodium dodecyl sulfate pre-heated at 70°C was added. Tubes were thoroughly vortexed for 5 s at maximum speed and inverted five times followed by 3 min at 4,000 rpm on a Bead Ruptor. The homogenate was then incubated at 70°C for 15 min with inversions every 5 min and centrifuged at 4°C for 15 min at 16,000 × *g*, and 900 µL of the supernatant was transferred to a new tube and then centrifuged at 4°C for 5 min at 16,000 × *g*. Approximately 800 µL of supernatant was transferred to a new tube. To maximize DNA recovery, resuspension buffer (800 μL), β-mercaptoethanol (5 μL), and 70°C pre-heated 10% sodium dodecyl sulfate (200 μL) were added to the remaining pellet and mixed, homogenized, and centrifuged to collect a second supernatant. The supernatants (lysates) from both homogenization steps were kept separate until column purification as described below. The lysates were mixed with 200 μL of 10 M ammonium acetate, kept on ice for 5 min, and centrifuged at 4°C for 10 min at 16,000 × *g*. The supernatants were mixed with an equal volume of isopropanol, placed on ice for 30 min, and centrifuged at 4°C for 15 min at 16,000 × *g*. Nucleic acid pellets were washed with 70% ethanol and dried before resuspension in 200 µL TE (10 mM Tris-HCl pH 7.4; 1 mM EDTA). To further purify metagenomic DNA, dissolved nucleic acids (200 μL) were mixed with 4 μL of DNase-free RNase (10 mg/mL) and incubated at 37°C for 15 min. Subsequently, 15 μL of proteinase K (20 mg/mL) and 200 μL of Buffer AL (QIAamp Fast DNA Stool Mini Kit; QIAGEN Inc., Toronto, ON, Canada) were added and incubated at 70°C for 10 min. Following incubation, absolute ethanol (200 μL) was added, and all the mixture was applied to the QIAamp column and centrifuged at 16,000 × *g* for 1 min. This step was repeated until all of the mixture of a sample was run through the same column. The column was washed with AW1 and AW2 buffers as per manufacturer’s instructions and dried by centrifugation. To elute DNA, pre-warmed (70°C) ATE buffer (50 μL, QIAamp Fast DNA Stool Mini Kit; QIAGEN Inc.) was added to the column and held at room temperature for 2 min followed by centrifugation. The elution step was repeated yielding a total volume of ~100 µL of DNA. Purity and concentration of DNA were quantified using a NanoDrop spectrophotometer (Thermo Fisher Scientific), while the integrity was verified by gel electrophoresis. Purified DNAs from the four blocks of a given sampling day and Zn supplementation combination were equally mixed prior to sequencing, resulting in a total of six metagenomes, each representing 10 animals (Fig. 1).

Libraries preparations and sequencing were conducted by the McGill University and Genome Quebec Innovation Centre (Montreal, QC, Canada). Illumina PCR-free shotgun DNA libraries were prepared as per manufacturer’s instructions. Samples were run on an Illumina NovaSeq 6000 S4 generating 150 bp paired-end (PE) sequences.

Raw reads were trimmed and decontaminated against a combination of the *Sus scrofa* (Ssrofa11.1), *Bos taurus* (ARS-UCD1.3), and phiX174 (NC_001422) genomes using the quality control modules of a Nextflow metagenomics pipeline named metagenomic_nf (https://github.com/AAFC-Bioinfo-AAC/metagenomic_nf) (Table S1). Taxonomy was inferred from shotgun sequencing data using the Kraken2 (v2.1.2) (42) and Bracken (v2.9) (43) modules of the Nextflow metagenomics pipeline metagenomic_nf. Using a confidence score of 0.5, reads were compared against the Kraken2 and Bracken database built with data from the Genome Taxonomy Database (GTDB r214) (44). For the detection of ARGs, filtered reads were analyzed with the AMR++ pipeline (v3.0.6) along with the MEGAres v3.0 database (https://github.com/Microbial-Ecology-Group/AMRplusplus) (45). Read counts were normalized using the cumulative sum scaling (CSS) method.

Downstream analyses were conducted in R (v4.3.1) using the R packages phyloseq v1.46.0 (46), vegan v2.6.8 (47), tidyverse v2.0.0 (48), and their dependencies. Visualizations were created in R using ggplot2 v3.5.1 (49) and Polychrome v1.5.1 (50).

### Amplicon sequencing

DNA extractions for 16S rRNA sequencing were performed on ileum and cecum samples using the Griffiths extraction protocol (51). Total DNA was purified using the Qiagen AllPrep DNA/RNA 96 kit according to manufacturer’s instructions. DNA was suspended in EB buffer and quantified with the Nanodrop 8000 and Quant-iT PicoGreen dsDNA Assay Kit (Life Technologies). Extracted DNAs were sent to Genome Quebec (Montreal, QC, Canada) for 16S rRNA amplicon preparation and sequencing (Fig. 1). Libraries were generated using the NEBNext Ultra II DNA Library Prep Kit for Illumina targeting the V4 region of 16S rRNA genes using the 515F/806R primer pair (52). Amplicon sequencing using the Illumina MiSeq technology generated 250 bp paired-end (PE) sequences. A phiX library was used as a control and mixed with libraries at 1% level. Fastq reads were processed with the nf-core/ampliseq pipeline v2.3.2 (53) for quality filtering, trimming, and taxonomic identification using the SILVA 138 database (54). Amplicon sequence variants with a frequency lower than 0.1% of the mean sample depth (cut-off of 99) were filtered out (https://github.com/LangilleLab/microbiome_helper/wiki/Amplicon-SOP-v2-[qiime2-2018.8]).

### High-Throughput qPCR

Prior to DNA extractions, equal volumes of colon digesta samples from two blocks fed the same Zn concentrations on day 42 were pooled, leading to six pools of five animals each. DNA was extracted using the same protocol as for shotgun metagenomics. Extracted DNAs for the four blocks of a given Zn group on day 42 were pooled and analyzed by high-throughput qPCR (HT-qPCR; Fig. 1).

Amplifications and quality control steps were conducted by Resistomap (Helsinki, Finland). Panel 2.1 was used to quantify 384 targets (Table S2) in the three pooled DNA samples. qPCR assays were done using a SmartChip (Takara) where 100 nL of each sample was distributed using a SmartChip Multisample Nanodispenser (Takara). Cycling conditions and initial data processing were conducted as described by Wang et al. (55), and qPCR reagents recommended by the manufacturer were used. Detection limit was set at a threshold cycle (Ct) of 27 (55–58). Abundances relative to the 16S rRNA gene were calculated using the 2^−∆Ct^ method for every detected gene in each sample (59). Assays were conducted in triplicate where the mean Ct of three technical replicates was used to calculate ∆Ct values.

### Droplet digital PCR

For droplet digital PCR (ddPCR) analysis, DNA extractions were conducted on each sample independently (*n* = 60) following the same protocol as for shotgun metagenomics. Extracted DNAs were pooled by block, sampling day, and Zn supplementation leading to 24 samples (Fig. 1). The ddPCR analyses were conducted by the RNomics Platform at the Université de Sherbrooke (Sherbrooke, QC, Canada). Reactions consisted of 10 µL of 2× QX200 ddPCR EvaGreen Supermix (Bio-Rad), 20 ng of DNA, and primer pair solutions (200 nM final). Primer pairs are listed in Table 1.

**TABLE 1:** Primer pairs used in the ddPCR assays

Gene	Primers (5′−3′)	Amplicon size (bp)	Reference
*16S rRNA*	AGAGTTTGATCMTGGCTCAG	~520	(60)
	GWATTACCGCGGCKGCTG		(61)
*bla*_CTX-M_	ATGTGCAGYACCAGTAARGTKATGGC	336	(62)
	ATCACKCGGRTCGCCNGGRAT		
*bla*_TEM_	GCATCTTACGGATGGCATGA	101	(63)
	GTCCTCCGATCGTTGTCAGAA		
*bla*_CMY-2_	AGACGTTTAACGGCGTGTTG	127	(64)
	TAAGTGCAGCAGGCGGATAC		
*intI1*	TCGTGCGTCGCCATCACA	67	(65)
	GCTTGTTCTACGGCACGTTTGA		
*sul1*	GACTGCAGGCTGGTGGTTAT	105	(66)
	GAAGAACCGCACAATCTCGT		
*sul3*	TCCGTTCAGCGAATTGGTGCAG	128	(67)
	TTCGTTCACGCCTTACACCAGC		
*tet*(Q)	AGGTGCTGAACCTTGTTTGATTC	69	(68)
	GGCCGGACGGAGGATTT		
*ermB*	AAAACTTACCCGCCATACCA	139	(69)
	TTTGGCGTGTTTCATTGCTT		
*sat4*	CTGCGAAAAAATTGGAACC	~238	(70)
	GTTGGCGTATAACATAGTATCG		
*zntA*	AGTACGTAACGATGACGTGCTTG	210	(71)
	GTCACCGCTTTGACTTTATCTTCC		
*pcoA*	GCTGCAGATGGCCAGTATGTAAA	147	(72)
	CCCTCGAGCGTAACCGGTCC		

Each reaction mix (20 µL) was converted to droplets with the QX200 droplet generator (Bio-Rad). Droplet-partitioned samples were then transferred to a 96-well plate and sealed and cycled in a C1000 deep well Thermocycler (Bio-Rad) under the following cycling protocol: 95°C for 5 min (DNA polymerase activation), followed by 50 cycles of 95°C for 30 s (denaturation), 59°C for 1 min (annealing), and 72°C for 30 s (extension) followed by post-cycling steps of 4°C for 5 min and 90°C for 5 min (signal stabilization) and an infinite 12°C hold. The cycled plate was then transferred and read using the QX200 reader (Bio-Rad) either the same or the following day post-cycling. The concentration was reported in gene copies/µL of the final 1× ddPCR reaction using the QuantaSoft software from Bio-Rad (73).

### Statistics

Measurements of pH and VFA in cecum and colon of piglets were compared between each concentration of Zn, before and after supplementation, based on a two-way analysis of variance (ANOVA) with Tukey’s multiple comparisons test. Alpha diversity indices in ileum and colon were computed using R package phyloseq v1.51.0 (46), and the results were also subjected to an ANOVA analysis using easyanova v11.0 (74).

The abundance table generated from the 16S rRNA sequencing data was subjected to Hellinger transformation, the data dispersion was assessed, and the beta diversity between feeding treatments was analyzed using a PERMANOVA followed by pairwise adonis comparison tests using the R packages vegan v2.6.10 (47) and pairwiseAdonis v0.4.1 (75). A custom R script was generated to determine discriminant bacterial families between the contrasting treatments in both ileal and cecal digesta. Given the limitations of discriminant analysis methods (DA) available (76), four DAs were compared: LEfSe, MaAsLin2, DESeq2, and ALDEx2. They were computed using R packages lefser v1.17.6 (77), Maaslin2 v.1.22.0 (78), DESeq2 v1.48.1 (79), ALDEx2 v1.40.0 (80), and their dependencies. Upset plots were created in R using the package ComplexUpset v1.3.3 (81, 82) to visualize consensus for the differential families between the DA tools.

Statistical analysis of ARGs quantified by ddPCR was conducted in SAS (83) with a complete random block design where a 3 × 2 factorial effect (three Zn supplementation, two sampling days) was used on six experimental units, each measured four times. The Kruskal-Wallis test was used to assess the variance between each Zn supplementation for both sampling days. Statistical significance was declared with a *P* < 0.05 for all analyses.

## RESULTS

### Gut microbiome

**Fig 2:**
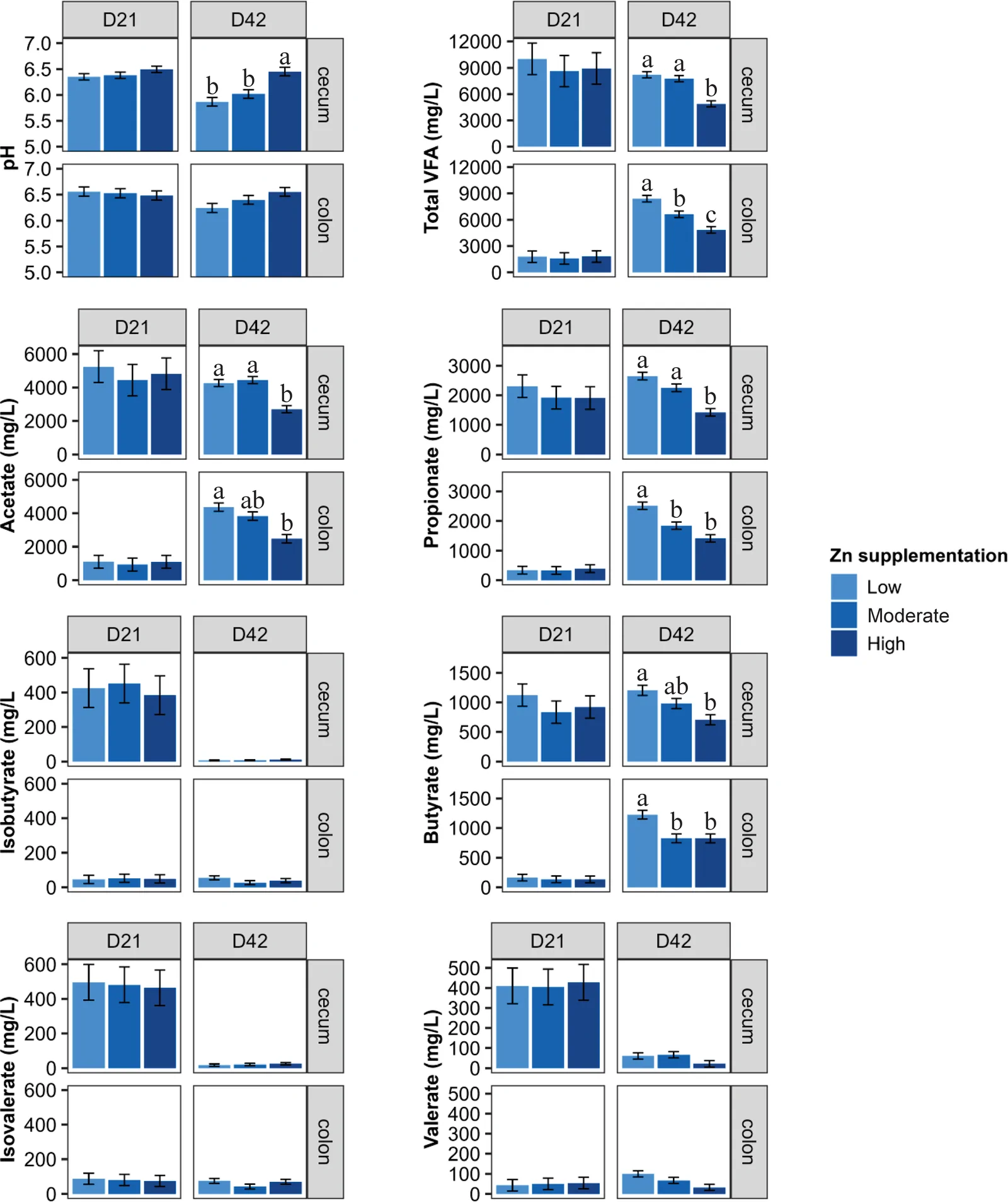
The pH and VFA concentrations in cecum and colon of piglets fed low, moderate, or high zinc diets. Bars represent adjusted means with confidence intervals. Different letters on top of the bars indicate significantly different results (*P* < 0.05) based on a two-way analysis of variance with Tukey’s multiple comparisons test.

To investigate the impact of Zn on the fermentative activity of gut microbial communities, the pH and VFA concentrations were measured in cecum and colon digesta of piglets at weaning prior to Zn supplementation (D21) and after 3 weeks of supplementation (D42). The cecal pH was higher in piglets fed with the high Zn diet, while it was more acidic when piglets were fed the low (*P* = 0.0003) or moderate (*P* = 0.005) Zn diets (Fig. 2). Although not statistically significant, these observations were also reflected in the colon (*P* = 0.0580). Conversely, the total concentration of VFA was lower in cecum and colon digesta of piglets fed with the high Zn diet (*P* < 0.0001). This observation was mainly due to the individual concentrations of acetate, propionate, and butyrate that were higher in the low Zn group compared to the high Zn in both the cecum and the colon after 3 weeks of Zn supplementation (*P* < 0.01).

**Fig 3:**
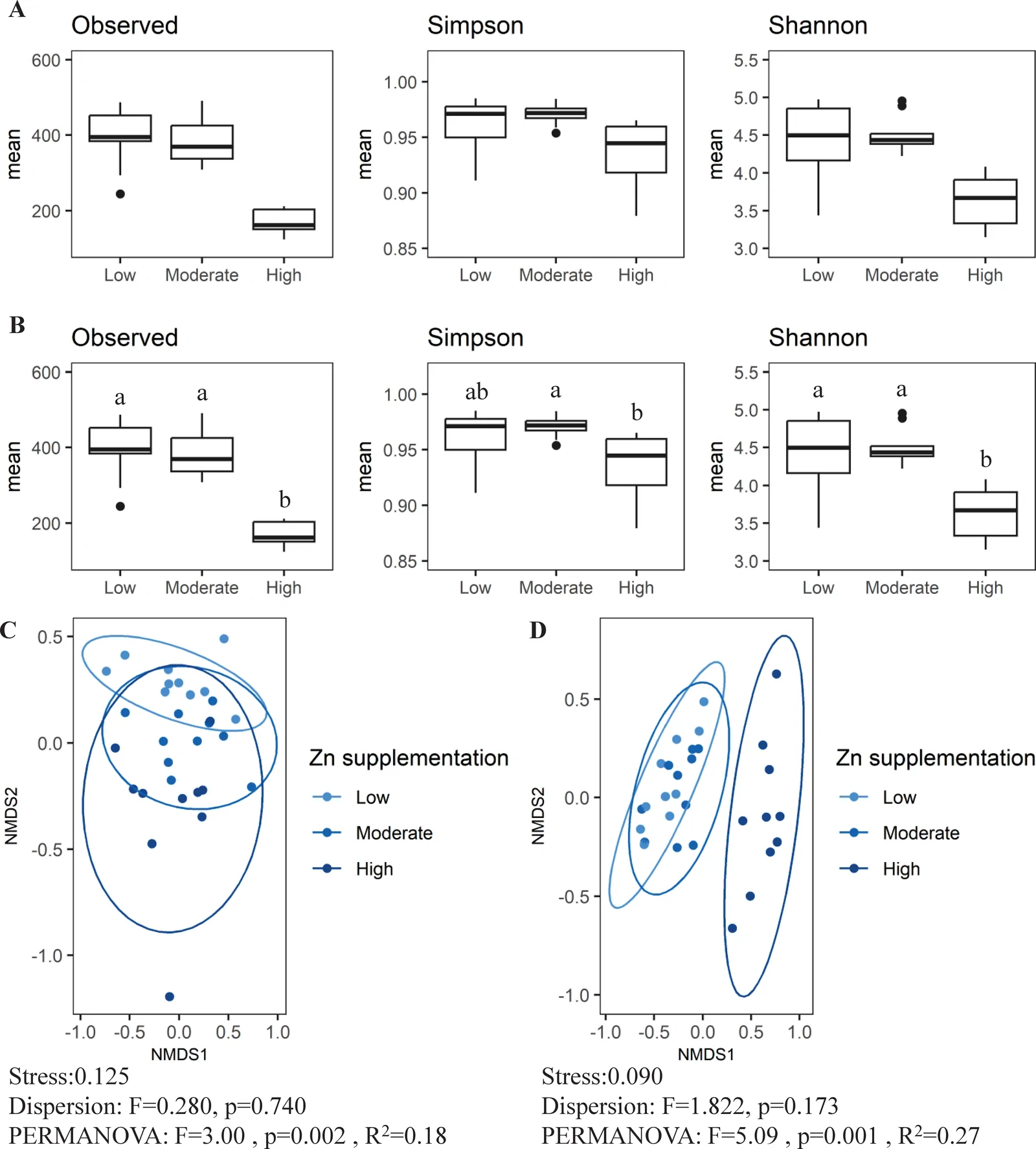
Diversity indices in digesta of piglets fed for 21 days with either low, moderate, or high zinc diet. Alpha diversity using Observed, Simpson, and Shannon indices in (**A**) ileum and (**B**) colon digesta. Beta diversity in (**C**) ileum and (**D**) colon digesta. Different letters on top of the boxplots indicate significantly different results (*P* < 0.05) based on pairwise adonis comparison tests.

**Fig 4:**
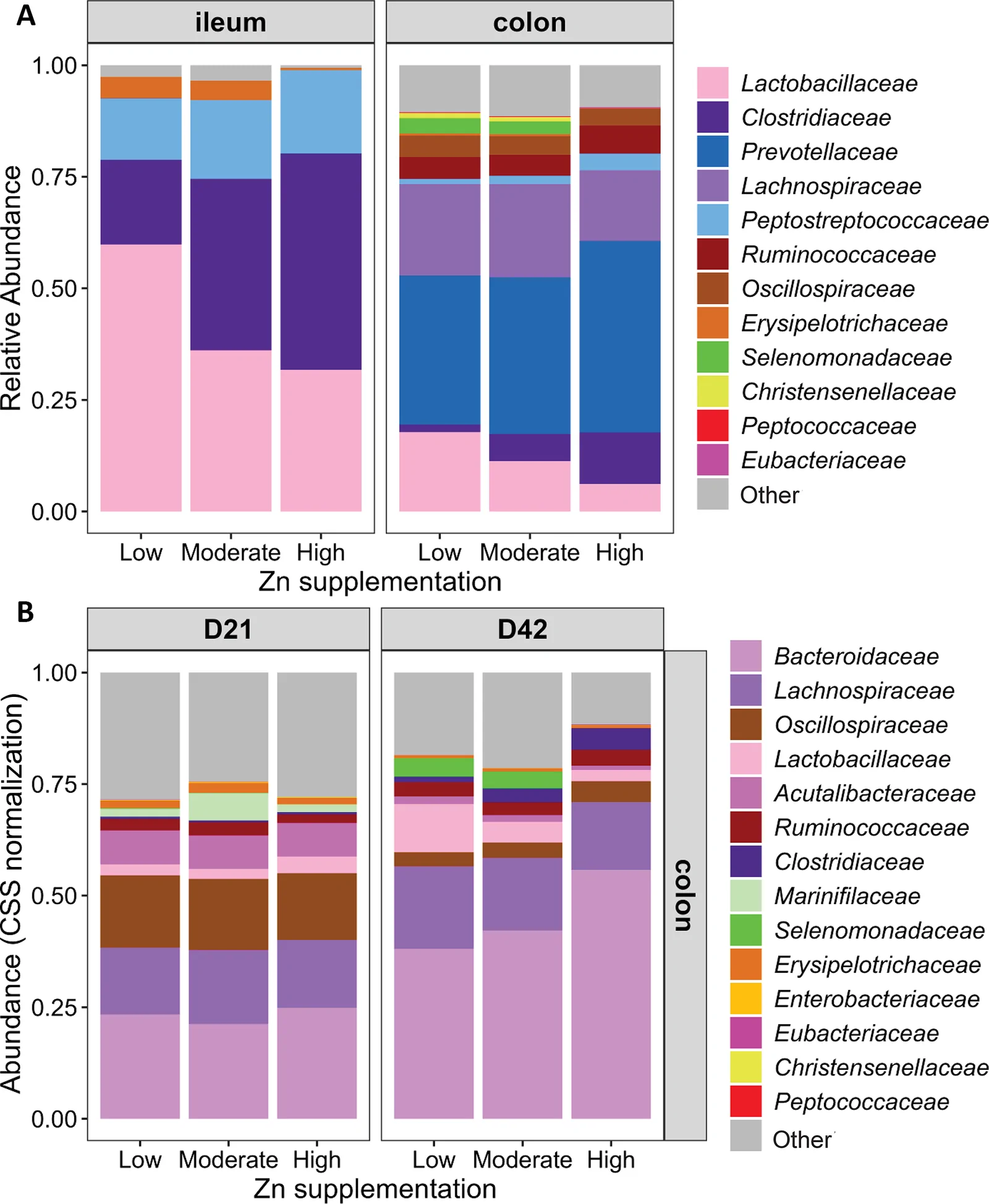
The bacterial families identified by (**A**) amplicon sequencing of ileal and colonic digesta of piglets subjected for 3 weeks to the low, moderate, or high zinc diets (D42) and (**B**) shotgun sequencing of colonic digesta prior (D21), and after (D42), 3 weeks of the contrasting in-feed zinc supplementations.

**Fig 5:**
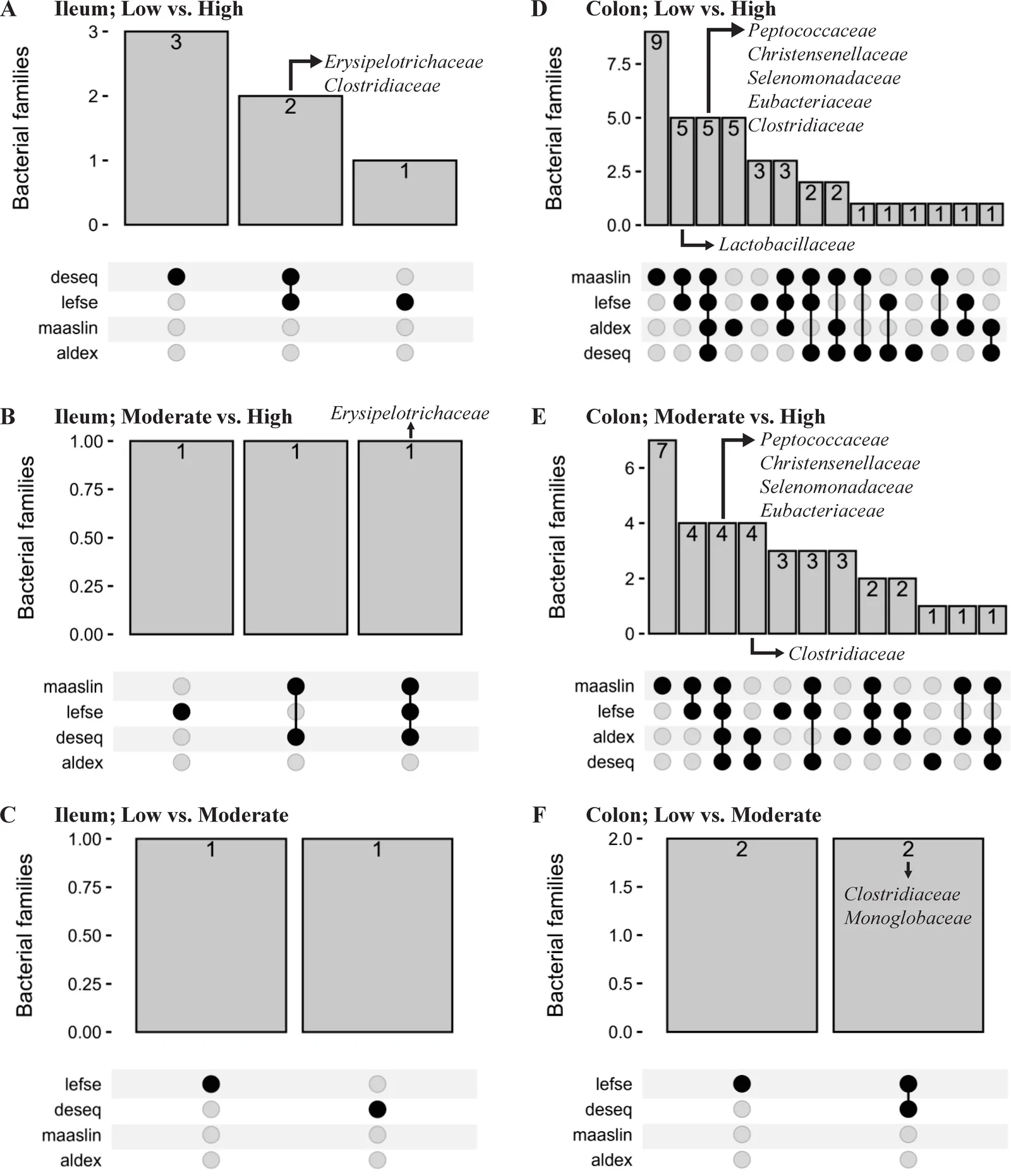
Bacterial families identified as discriminant using MaAsLin2 (maaslin), LEfSe (lefse), ALDEx2 (aldex), and DESeq2 (deseq) analyses in ileum of piglets fed (**A**) low vs high Zn, (**B**) moderate vs high Zn, (**C**) low vs moderate Zn and in colon of piglets fed (**D**) low vs high Zn, (**E**) moderate vs high Zn, (**F**) low vs moderate Zn diets. Taxa with consensus and/or consistent with relative abundance data are indicated.

Two complementary sequencing methods were used to investigate the impact of contrasting Zn dosages in diet on the composition of the piglet gut microbial communities. Amplicon sequencing was used with a sufficient number of samples to reach statistical power, while a limited number was analyzed by shotgun metagenomics to validate the microbial communities observed. Rarefaction curves with both methods confirmed that the sequencing depth was sufficient for a good representation of the taxa as they reached a plateau for all samples (data not shown). The abundances of taxa identified by amplicon sequencing are provided in Table S3. It revealed that the alpha diversity of the microbial communities in the ileum of piglets was not significantly impacted by the treatments (Fig. 3A). However, the diversity was reduced in the colon of piglets fed with the high Zn diet (Fig. 3B). The bacterial communities in both the ileum (Fig. 3C; *P* = 0.009) and colon (Fig. 3D; *P* = 0.003) of high Zn-fed piglets were altered in comparison to those fed with the low Zn diet. Ileum samples after the Zn treatments were mostly composed of *Lactobacillaceae*, *Clostridiaceae,* and *Peptostreptococcaceae* families, whereas *Prevotellaceae*, *Lachnospiraceae,* and *Lactobacillaceae* were the main families in colon samples (Fig. 4A). The bacterial families in colon digesta were more diverse than in ileum digesta and included larger proportions of beneficial anaerobes including *Lachnospiraceae* and *Ruminococcaceae* (Fig. 4A). Both sequencing methods revealed that *Prevotella* dominated the colon bacterial populations and increased with the Zn concentration. Although, the SILVA 138 database used for amplicon sequencing categorized *Prevotella* in *Prevotellaceae* (Fig. 4A), while the MEGAres database categorized them as *Bacteroidaceae* (Fig. 4B). Both discriminant DESeq2 and LEfSe analyses showed that the proportions of *Clostridiaceae* and *Erysipelotrichaceae* were significantly different between the ileum of piglets fed with the low or the high Zn diets (Fig. 5A). *Clostridiaceae*, mainly composed of the genus *Clostridium sensu stricto 1*, increased, while the *Erysipelotrichaceae*, mostly composed of the genus *Turicibacter*, decreased in the ileum of piglets fed the high Zn diet in comparison to the low Zn diet (Fig. 4A). Coherently, *Erysipelotrichaceae* were also discriminant between the moderate and high Zn groups (Fig. 5B), for which higher levels were seen in the ileum of piglets fed the moderate Zn diet. No consensus between DAs was obtained when comparing bacterial families in the ileum of piglets fed either low or moderate levels of Zn (Fig. 5C). In the colon, the LEfSe and MaAsLin2 discriminant analyses revealed that *Lactobacillaceae* were less abundant in the colon of high Zn fed piglets than in the colon of piglets fed low concentrations of Zn (Fig. 5D). All four discriminant analyses revealed that the relative abundances of *Peptococcaceae*, *Christensenellaceae,* and *Selenomonadaceae* were lower in high Zn fed piglets, whereas *Eubacteriaceae* were more abundant (Fig. 5D and E). *Clostridiaceae* were also more abundant in colons of piglets fed the high Zn diet than of the others (Fig. 5D and E). Fewer bacterial families found in the colon were discriminant between piglets fed the low or moderate Zn diets (Fig. 5F). Both sequencing methods revealed that the proportions of *Clostridiaceae* and *Lactobacillaceae*, dominated by the genus *Lactobacillus*, were, respectively, proportional and inversely proportional to the concentration of Zn in the diet (Fig. 4A and B). Both amplicon and shotgun sequencing also revealed a sharp depletion of *Selenomonadaceae* in colonic digesta of piglets fed with high Zn levels (Fig. 4A and B).

Regardless of the treatment, bacterial populations shifted between the two sampling days, where *Oscillospiraceae* and *Acutalibacteraceae* were proportionally more abundant at weaning than 3 weeks later (Fig. 4B). On the other hand, *Clostridiaceae* and *Bacteroidaceae*, mostly composed of the genus *Prevotella*, accounted for a larger fraction of the microbiome on day 42 than on day 21. The proportion of *Lactobacillaceae* increased with age for the low and moderate Zn diets, but not for the high Zn diet.

### Gut resistome

**Fig 6:**
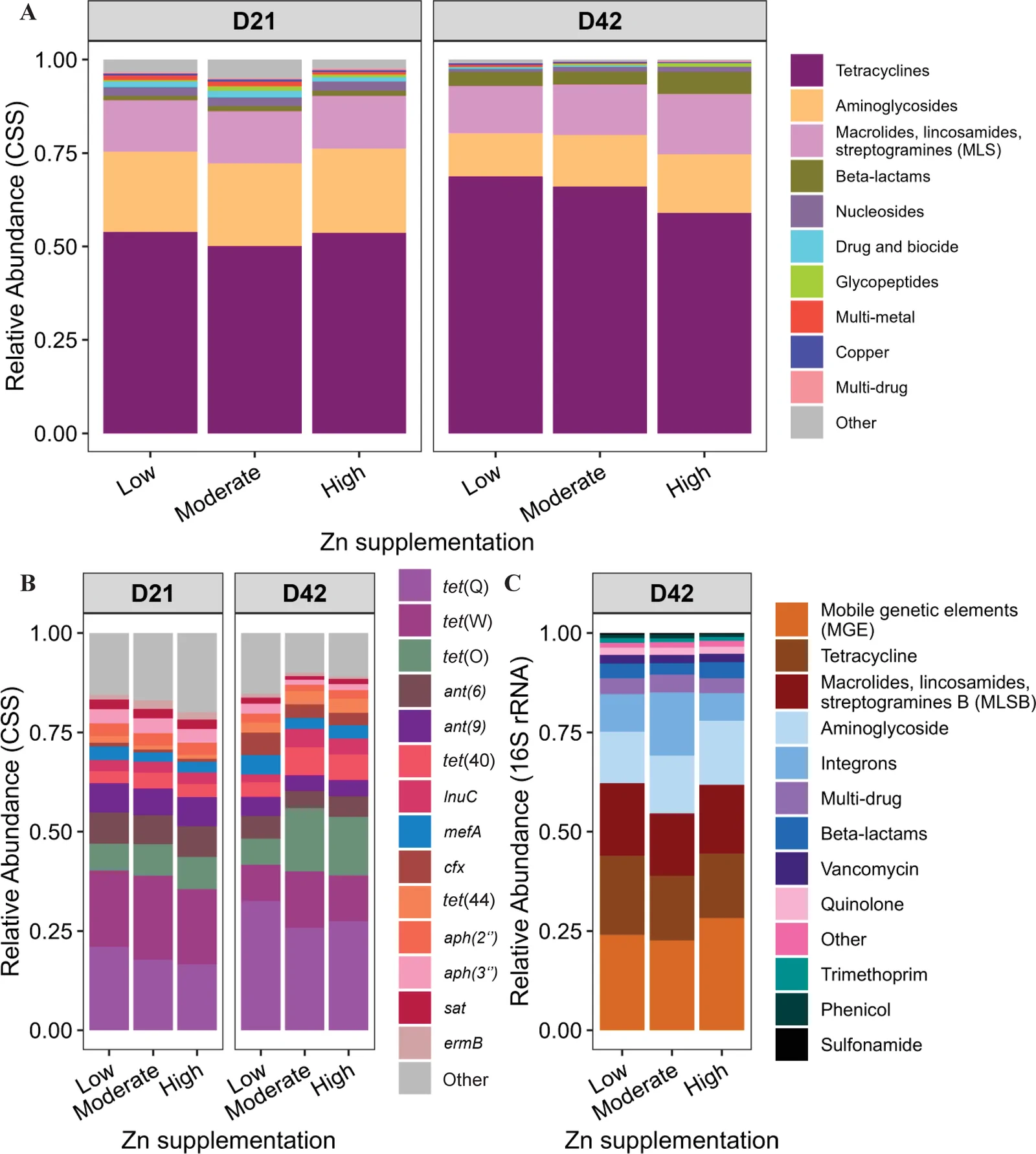
Resistome in colonic digesta of piglets prior (D21) or after (D42) 3 weeks of consumption of low, moderate, or high zinc diets. Shotgun metagenomics provided (**A**) relative abundances normalized using cumulative sum scaling (CSS) of antibiotic resistance genes grouped in classes according to the resistance they confer and (**B**) relative abundances (normalized with CSS) of individual antibiotic resistance genes. HT-qPCR supplied (**C**) relative abundances of 375 resistance genes and mobile genetic elements normalized with the 16S rRNA gene and grouped according to the resistance they confer.

The gut resistome was profiled using a combination of shotgun metagenomics, HT-qPCR, and ddPCR to capture the resistome broadly and confirm specific targets. First, metagenomic analyses were performed on colonic digesta before and after the administration of the contrasting Zn diets. Rarefaction curves confirmed that a depth sufficient for a good representation of ARGs was reached for all samples (data not shown). Tetracycline resistance genes were the most prevalent genes recovered in all samples (Fig. 6A), specifically, *tet*(Q), *tet*(W), and *tet*(O) (Fig. 6B). At day 21, variations between groups were notable even though animals were not yet subjected to the treatments. In all animal groups, the proportion of genes conferring resistance to tetracyclines and β-lactams was higher at 42 days of age, while we observed a consistent reduction in aminoglycosides resistance genes from days 21 to 42 (Fig. 6A). Moreover, the resistome appeared slightly more diverse on day 21 than on day 42. β-Lactams appeared enriched in microbiomes exposed to high concentration of zinc.

As a preliminary assay to confirm the presence of ARGs, a panel of 375 resistance genes and markers of mobile genetic elements were quantified by HT-qPCR in colonic digesta of piglets subjected to the contrasting Zn diets. Patterns of ARG groups were similar between the three animal groups, for which the most frequently encountered genes were coding for mobile genetic elements and integrons, as well as by genes conferring resistance to tetracyclines, macrolides, lincosamides and streptogramins B, and aminoglycosides (Fig. 6C).

**Fig 7:**
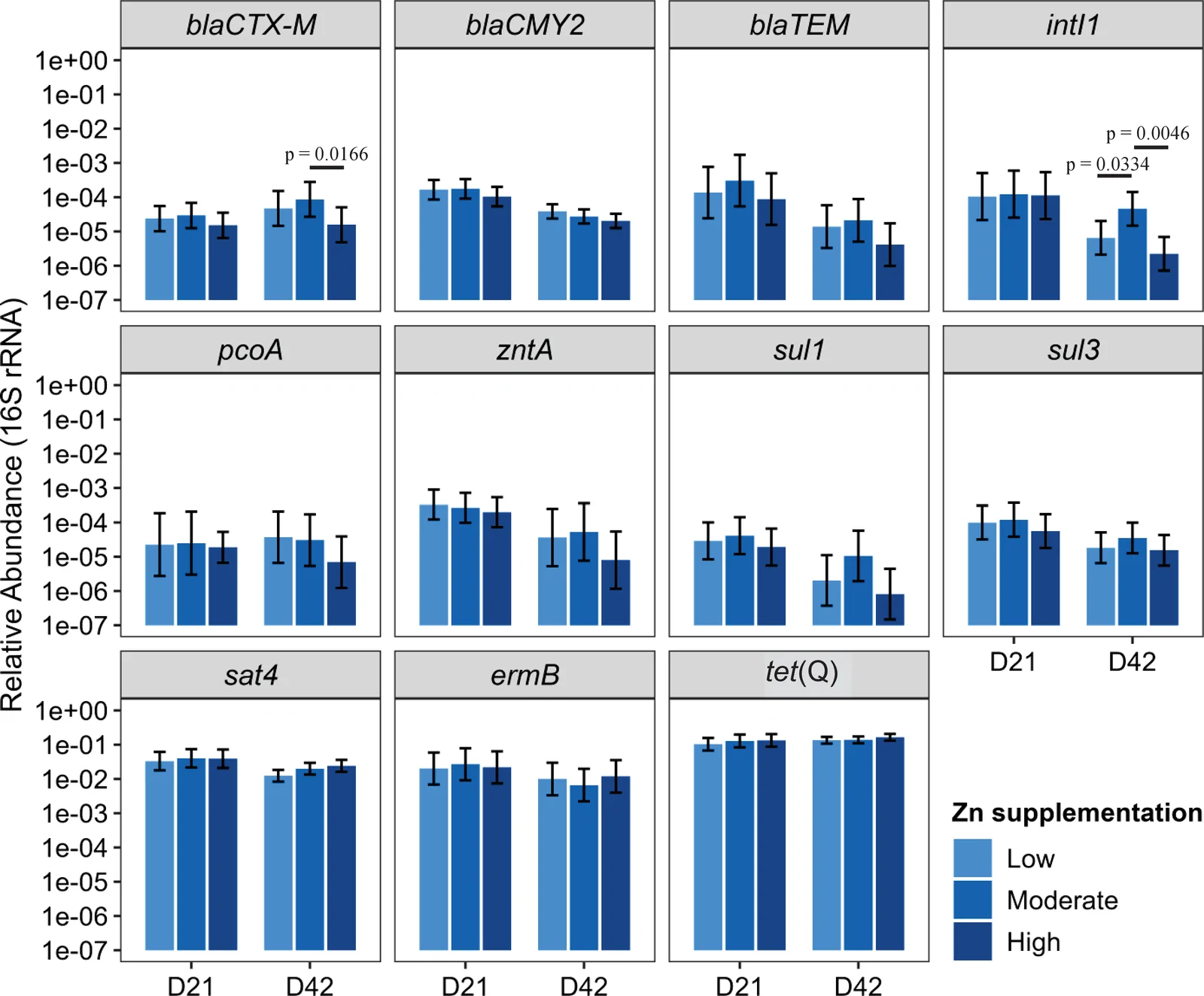
Quantification of antibiotic resistance genes using ddPCR in colon samples. Genes were quantified before (D21) and after 21 days of zinc supplementation (D42). The bars represent the ls means. The error bars represent the upper and lower bounds of the confidence interval. Logarithmic transformations of relative abundance of ARGs quantified by ddPCR were analyzed. The Kruskal-Wallis test was used to assess the variance between each Zn supplementation for the two sampling days.

Based on the results obtained by shotgun metagenomics and HT-qPCR (Table 2), 12 specific genes (Table 1) were quantified using ddPCR in composite colonic digesta samples, each representing two or three animals, prior and after the administration of the treatments. Kruskal-Wallis tests revealed an impact of the treatments on the abundance of the *bla*_CTX-M_ (*P* = 0.0498), *intI1* (*P* = 0.0183), and *sat4* (*P* = 0.0366) genes. After 3 weeks of treatment, the abundance of *bla*_CTX-M_ was higher in the moderate Zn group than in the high Zn group (Fig. 7). Similarly, the abundance of *intI1* was also greater in the moderate Zn group at day 42. The relative abundances of the other targeted genes were not significantly affected by the treatments according to the conducted Kruskal-Wallis statistical analysis.

**TABLE 2:** Major alleles quantified by shotgun metagenomics and HT-qPCR classified according to the resistance they confer

	Shotgun metagenomics		HT-qPCR	
	Antimicrobial class	Alleles	Antimicrobial class	Alleles
Antibiotic resistance	Tetracycline	*tet*(Q), *tet*(W), *tet*(O), *tet* (40)	Tetracycline	*tet*(Q), *tet*(W), *tet*(D), *tet*(O)
	Aminoglycoside	*ant6, ant9*	Aminoglycoside	*aph3-iii, ant6-ia, aadA7*
	MLS	*lnuC, mefA*	MLSB	*lnuC, mefA, mphA*
	β-Lactam	*cfx*	β-Lactam	*blaGOB, blaSFO, blaOXY, cfxA, penA, blaCTX-M*
	Glycopeptide	*vanG*	Glycopeptide	*vanA*
			Fluoroquinolone	*qepA*
	Diaminopyrimidine	*dfrF*	Diaminopyrimidine	*dfra21, dfrA25, dfrA27, drA7*
	Phenicol	*catS*	Phenicol	*cmIV*
	Sulfonamide	*sul2*	Sulfonamide	*sul3*
	Nucleoside	*sat*		
	Rifamycin	*rpoB*		
	Nitroimidazole	*nimA, nimF, nimJ, nimH*		
	Peptide	*bacA*		
Multi-drug,	Multi-drug	*lsaC, msbA, lsaE*	Multi-drug	*oprD, sugE*
Metal and biocide resistance	Multi-metal	*cusA, zntA, pitA*		
	Drug and biocide	*acrB, mdtF*		
	Biocide and metal	*sitABCD*		
	Drug and biocide and metal	*mdtC, mdtB, acrD*		
	Iron	*ybtP, ybtQ*		
	Multi-drug	*lpdT*		
	Nickel	*nikA, nikC*		
	Zinc	*znuC*		
	Copper	*tcrB*		
	Arsenic	*pstB*		
	Multi-biocide	*yjcG*		
	Sodium	*nhaB, chaA, nhaA*		
	Peroxide	*sodA*		
	Chromium	*nfsA*		
	Mercury	*merC*		
MGE			MGE	*IS613, Tp614*
Integrons			Integrons	*intI3, intI1*
Other	Cationic antimicrobial peptides	*eptA, ugd, mprF*	Other	*sat4, bacA*
	Acid	*gadA*		
	Phenolic compound	*fabI*		

## DISCUSSION

The inclusion of supranutritional concentrations of Zn in post-weaning piglet diets is a common practice in countries that are among the leading exporters of pork, such as Canada (20), USA, Brazil, and China (7, 8, 84, 85). It is used as an alternative or as a complement to preventive antibiotic treatments to maintain piglets' health during weaning and mitigate the associated economic loss. Feeding high Zn concentrations to pigs in commercial settings poses environmental issues and is also of concern in the fight against AMR as it can act as a selecting agent. The aim of this study was to evaluate the impact, on the gut microbiome and resistome, of three concentrations of ZnO fed to post-weaning piglets in controlled conditions. This study is complementary to the physiological and metabolic analyses performed on the same animals by collaborators (35–37) and revealed that a high Zn diet negatively impacted *Lactobacillus*, a recognized probiotic (86), as well as other beneficial bacteria, namely, *Peptococcaceae*, *Christensenellaceae, Selenomonadaceae*. They are at the core of the fermentative ability of the pig gut microbiome as they are key producers of short-chain fatty acids, are important for feed efficiency and weight gain, and ultimately contribute to a healthy gut development (87, 88).

Coherent with previous literature (23, 89–91), age and diet impacted the gut microbiome of piglets. De Rodas et al. (92) investigated the gut microbiome of pigs from farrow to finish and concluded that the switch of diet (milk to solid) had the greatest impact. Our results are also similar to observations by Guevarra et al. (91), where *Prevotella* and *Lactobacillus* were enriched in weaned compared to nursed piglets, and seem to be linked to dietary changes as the microbiota has to adapt from a milk-oriented to a solid diet (93). Indeed, *Prevotella* can degrade fiber from plant polysaccharides, which could explain their increase when piglets were switched to the plant-based diet (94). On the other hand, some families were decreased after weaning. *Oscillospiraceae* are known to be negatively affected by Cu (95) which is also commonly administered orally at high concentrations to post-weaning piglets in Canada (20), including in this study, where all the diets contained 126 ppm of copper sulfate.

The bacterial diversity of ileal communities was not altered by 3 weeks of contrasting Zn supplementations. However, higher abundance of *Clostridiaceae* and lower abundance of *Erysipelotrichaceae* were observed when piglets were fed the high Zn diet in comparison to those fed the low Zn diet. Other studies found stronger correlations between ZnO supplementation and ileal bacterial alterations (96, 97). Differences could be attributed to variability between herds, concentrations of ZnO fed as well as weaning age.

Moving on to the cecum, piglets fed with the high Zn concentration showed higher pH, as well as lower VFA concentrations, particularly acetate, propionate, and butyrate. The same results were observed in the colon. An increase in cecal pH and a reduction in VFA production throughout the gut were also observed by Hojberg et al. (98) in which pigs were supplemented with 2,500 ppm of ZnO in contrast to 100 ppm. The observed negative correlation between gut pH and VFA levels is in accordance with the literature (99). Acetate, propionate, and butyrate are known to be the main VFAs produced in the colon through fermentation of dietary fibers (100). Production of VFA is positive from a nutritional standpoint as complex fibers become available for bacteria. It is also of importance for host health and immune function (100). For example, their production acidifies the gut environment, which can limit the establishment of pathogens, including *Enterobacteriaceae*. This process, called lactic fermentation, is widely used to produce fermented foods and limit the growth of food spoilage bacteria (101). Modifications in environmental pH alter the growth of the microbial communities in this same environment, as some bacteria are more tolerant to lower pH, for instance, *Firmicutes* and *Actinomycetes,* while others like *Bacteroides* are more sensitive (102).

In contrast to the ileum, bacterial diversity was depleted by the high concentration of Zn in the colon digesta. In comparison to piglets fed low levels of Zn, those fed high levels showed an increase in *Clostridiaceae* and a decrease in *Lactobacillaceae* in their colon. A decrease in *Lactobacillus* was also observed in the high zinc treatment of a study comparing 57 ppm of ZnO with 2425 ppm (103), which was also reflected in other studies (104). Even though the mechanisms are still not fully understood, *Lactobacillaceae* seem to be impacted by metal availability, including Zn (104). Even though changes in the microbiome are not consistent between all the studies, most likely due to inherent differences between herds, the decrease in *Lactobacillus* is often observed (105), which was the dominant genus of *Lactobacillaceae* found in colon digesta. As *Lactobacillus* are known to be beneficial and often used as probiotics (106), their decrease is thought to be detrimental to gut health. On the other hand, an increase in *Clostridiaceae* can be seen negatively as some are pathogenic although not all species are. The predominant genus in this study was *Clostridium sensu stricto 1*, which does include some pathogenic species (107). Therefore, this increase in *Clostridiaceae* could be detrimental for the animals. Drake et al. (108) also studied the impact of overfeeding Zn, but on the fecal microbiome of gestating sows. They found that feeding 2,500 ppm of Zn led to an increase of unclassified *Clostridiaceae* as well as a decrease in *Turicibacter* and unclassified *Christensenellaceae* in comparison to those fed with 125 ppm of Zn.

Overall, Zn supplementation had a different impact on the ileum, cecum, and colon. As they have different functions, different responses to Zn supplementation in the gut were to be expected. This difference between gut sites is likely due to their distinct composition as the ileal microbiota is generally less rich and diverse than the highly fermentative microbiota of the colon (100, 109). As shown in other studies, it could be hypothesized that ZnO has a bacteriostatic effect (98, 103) although this effect cannot be generalized to an entire bacterial family and is rather species specific (110). Even so, this antibacterial activity can lead to a decrease in VFA production and ultimately to an increase in pH. As already mentioned, pH alteration can favor or deplete some bacteria, modulating the microbiome.

In addition to the microbiome analysis, the resistome was also assessed in the current study. As expected, tetracycline resistance genes were predominant, which is consistent with other studies (111, 112). For decades, tetracyclines were used profusely in agriculture, leading to the solid embedment of resistance against them in livestock gut microbiomes, particularly in swine (113).

The resistomes were more diverse at weaning than 3 weeks later, which was also observed by Gaire et al. (114) and others (89, 115). This evolution of the resistome is most likely linked to microbiome dynamics, as some bacterial families like the *Enterobacteriaceae*, which are known to harbor ARGs, decrease with age (116), which is also observed in humans (117). Surprisingly, a higher abundance of *bla*_CTX-M_ and *intI1* genes was observed in the colon of piglets fed with moderate Zn levels than with high Zn concentrations. These results support that the moderate Zn treatment favored the *intI1* encoding bacteria, consistent with previous work (118), but that Zn included in feed at 2,991 ppm exhibited an observable antibacterial activity against the bacteria carrying *bla*_CTX-M_ and *intI1*.

Based on shotgun metagenomics, HT-qPCR and ddPCR results, differential Zn feeding had little impact on the gut resistome of weaned piglets although predominant genes conferring resistance to each AMR class were different between the metagenomics and the HT-qPCR. Furthermore, tetracycline resistance genes were not as predominant when looking at HT-qPCR results compared to metagenomics. On day 42, all dietary treatments put together, they accounted for 66% in metagenomes and 17% in HT-qPCR in piglets' colon. Those differences could be attributed to the techniques themselves as HT-qPCR relies on amplification, which was not the case for shotgun metagenomics as it was PCR-free, as well as on the databases used for identification. The MEGAres v3.0 database was used to analyze the metagenomes, which out of the 1,161, contains 60 genes conferring resistance to tetracyclines, whereas the panel used for HT-qPCR contained 26 tetracycline resistance genes out of the 384 tested. There were 21 *tet* genes common to both methods, while 5 were exclusive to HT-qPCR and 33 exclusive to MEGAres v3.0.

### Conclusion

This microbiome and resistome investigation was designed as a transdisciplinary complement to the in-depth study of physiological impacts of various Zn concentrations in piglets (35, 36). Many observations of microbial dynamics were observed with both sequencing methods despite the differences in the DNA extraction and taxonomy annotation methods as well as the number of samples analyzed. Moreover, three complementary techniques (metagenomics, HT-qPCR, ddPCR) were used to thoroughly assess the resistome.

The microbiome seemed more impacted by the dietary Zn supplementation than the resistome after 21 days of feeding piglets with discriminant diets. Indeed, feeding high levels of Zn to piglets appears to be detrimental for the gut microbiome since it led to an increase in pH, a decrease in VFA accumulation, as well as a decrease in the bacterial diversity, associated with increased levels of *Clostridiaceae* and a decrease of the *Lactobacillaceae*. Nevertheless, piglets fed moderate concentration of Zn showed higher abundance of *bla*_CTX-M_ and *intI1*. The results gathered in this study indicate that dietary Zn fed to post-weaning piglets should be limited to low levels as it negatively impacts the fermentative capacity of the gut microbiota. Further investigations are needed to fully understand the underlying mechanisms responsible for the detrimental impacts of feeding Zn (and other trace minerals) at supranutritional levels on the pig gut resistome as well as its environmental implications.

## Supplementary Material

Reviewer comments

## Data Availability

DNA sequences from both shotgun and amplicon sequencing have been deposited to NCBI and are accessible under accession number PRJNA1337434.

## SUPPLEMENTAL MATERIAL

Table S1 spectrum.00059-26-s0001.docx Number of reads per metagenome after each quality filtering step.

Tables S2 and S3 spectrum.00059-26-s0002.xlsx Table S2: Primers used by Resistomap. Table S3: Abundances of taxa identify by 16S rRNA amplicon sequencing.

OPEN PEER REVIEW reviewer-comments.pdf An accounting of the reviewer comments and feedback.

ASM does not own the copyrights to Supplemental Material that may be linked to, or accessed through, an article. The authors have granted ASM a non-exclusive, world-wide license to publish the Supplemental Material files. Please contact the corresponding author directly for reuse.
