# Supplementary material for: Characterization of the gut microbiome and resistome of piglets supplemented with low, moderate, and high concentrations of zinc oxide
Source: Microbiol Spectr. 2026 Sep 3;14(10):e00059-26. doi: 10.1128/spectrum.00059-26 (PMC13689069; doi:10.1128/spectrum.00059-26)
Supplement: Supplementary file 1 [file reviewer-comments.pdf]

ASM | Microbiology Spectrum

Characterization of the gut microbiome and resistome of piglets supplemented with low, moderate and high concentrations of zinc oxide

Alexandra Langlois, Antony Vincent, Karoline Lauzon, Jean-Simon Brouard, Danyel Bueno Dalto, Nathalie Gagnon, Guylaine Talbot, Jérôme Lapointe, and Dominic Poulin-Laprade

Corresponding Author(s): Dominic Poulin-Laprade, Agriculture and Agri-Food Canada

Review Timeline:

Submission Date:	January 9, 2026
Editorial Decision:	May 2, 2026
Revision Received:	June 22, 2026
Accepted:	July 31, 2026

Editor: Samara Mattiello

Reviewer(s): Disclosure of reviewer identity is with reference to reviewer comments included in decision letter(s). The following individuals involved in review of your submission have agreed to reveal their identity: Guangliang Gao (Reviewer #2); Festus Adeyemi Adejoro (Reviewer #3)

Transaction Report:

(Note: With the exception of the correction of typographical or spelling errors that could be a source of ambiguity, letters and reports are not edited. The original formatting of letters and referee reports may not be reflected in this compilation.)

DOI: https://doi.org/10.1128/spectrum.00059-26

Re: Spectrum00059-26 (Characterization of the gut microbiome and resistome of piglets supplemented with low, moderate and high concentrations of zinc oxide)

Dear Dr. Dominic Poulin-Laprade:

Thank you for the privilege of reviewing your work. Below you will find my comments, instructions from the Spectrum editorial office, and the reviewer comments.

Please return the manuscript within 60 days; if you cannot complete the modification within this time period, please contact me. If you do not wish to modify the manuscript and prefer to submit it to another journal, notify me immediately so that the manuscript may be formally withdrawn from consideration by Spectrum.

Revision Guidelines
To submit your modified manuscript, log into the submission site at https://spectrum.msubmit.net/cgi-bin/main.plex. Go to Author Tasks and click the appropriate manuscript title to begin. The information you entered when you first submitted the paper will be displayed; update this as necessary. Note the following requirements:

• Upload point-by-point responses to the issues raised by the reviewers in a file named "Response to Reviewers," NOT in your cover letter.
• Upload a compare copy of the manuscript (without figures) as a "Marked-Up Manuscript" file.
• Upload a clean .DOC/.DOCX version of the revised manuscript and remove the previous version.
• Each figure must be uploaded as a separate, editable, high-resolution file (TIFF or EPS preferred), and any multipanel figures must be assembled into one file.
• Any supplemental material intended for posting by ASM should be uploaded with their legends separate from the main manuscript. You can combine all supplemental material into one file (preferred) or split it into a maximum of 10 files with all associated legends included.

For complete guidelines on revision requirements, see our Submission and Review Process webpage. Submission of a paper that does not conform to guidelines may delay acceptance of your manuscript.

Data availability: ASM policy requires that data be available to the public upon online posting of the article, so please verify all links to sequence records, if present, and make sure that each number retrieves the full record of the data. If a new accession number is not linked or a link is broken, provide Spectrum production staff with the correct URL for the record. If the accession numbers for new data are not publicly accessible before the expected online posting of the article, publication may be delayed; please contact production staff (Spectrum@asmusa.org) immediately with the expected release date.

Publication Fees: For information on publication fees and which article types are subject to charges, visit our website. If your manuscript is accepted for publication and any fees apply, you will be contacted separately about payment during the production process; please follow the instructions in that e-mail. Arrangements for payment must be made before your article is published.

ASM Membership: Corresponding authors may join or renew ASM membership to obtain discounts on publication fees. Need to upgrade your membership level? Please contact Customer Service at Service@asmusa.org.

The ASM Journals program strives for constant improvement in our submission and publication process. Please tell us how we can improve your experience by taking this quick Author Survey.

Thank you for submitting your paper to Spectrum.

Sincerely,
Samara Mattiello
Editor
Microbiology Spectrum

Reviewer #2 (Comments for the Author):

The manuscript provides valuable insights into the effects of zinc supplementation on the piglet gut microbiome. While the study shows promise, a major revision is required to improve clarity, strengthen the methodological rigor, and deepen the analysis. A more detailed explanation of the methodology, coupled with additional analyses, will enhance the manuscript and provide more compelling evidence regarding the effects of zinc supplementation on the microbiome of piglets.

1 There is a discrepancy in the reported "high zinc" concentrations. In the Abstract (line 22), it is stated as 2291 ppm, while in the Methods section (lines 108 and 458), it is listed as 2991 ppm. This inconsistency needs to be corrected.
2 The rationale behind the selection of zinc concentrations (low, moderate, high) is not adequately explained. The authors should clarify whether these concentrations reflect industry practices or are based on previous literature. Furthermore, it would be useful to reference regulatory standards to justify the high zinc levels used.
3 While pH and VFA are evaluated, additional physiological and immunological assessments (e.g., serum zinc levels, immune responses) should be included for a more comprehensive evaluation of the health impacts of zinc supplementation.
4 The authors should provide justification for this timeframe or consider extending it to assess the long-term effects of zinc exposure on piglet growth, immunity, and microbiota. Including different growth stages would provide a better understanding of the long-term consequences.
5 In lines 192-194, the authors should describe their data preprocessing steps (e.g., quality control, contamination removal, normalization). Multivariate analyses such as redundancy analysis (RDA) or PCA should be used to assess the impact of zinc on microbiota, and more sophisticated statistical models (e.g., structural equation modeling) could help explore causal relationships. Data visualization could be enhanced with heatmaps, box plots, and violin plots, and p-value corrections (e.g., FDR, Bonferroni) should be applied to ensure the robustness of the results.
6 detailed tables presenting microbial abundance data across different groups should be provided to enhance transparency and assist future replication of the study.

Reviewer #4 (Comments for the Author):

This study addresses an important and timely topic in swine nutrition and antimicrobial resistance management by investigating the effects of dietary zinc oxide supplementation on gut microbial community composition and antibiotic resistance genes in the ileum and colon. This research may have significant implications for improving antimicrobial stewardship in animal production systems while maintaining animal health.
However, I have identified several areas that require clarification and improvement before the manuscript can be considered for publication.

Characterization of the gut microbiome and resistome of piglets supplemented with low, moderate and high concentrations of zinc oxide

Specific comments:

Abstract

L29-30: Is this a positive or negative outcome? That context will be good in the abstract.

Keywords: Replace microbiome and resistome in the keyword. They already appear in title.

Introduction

L56-57: aside mortality, the significance of post weaning growth depression and morbidity is huge from the economic and animal welfare perspective. Re-phrase.

L64: 100 ppm of Zn per day? Per kg of feed intake? Specify.

L105,229. Remove the initials from D.B Dalto et al. and F.H Wang

L115-117. The sentences here need re-written for clarity. Measurement of pH for colon and cecum; was this done on digesta sample collected before dilution? What quantity of digesta was sampled, with which equipment/device.

L146: 0.3 g of 0.1 mm….?

L428-430. Recast this sentence. Which of the ZnO levels decreased Lactobacillu57 or 2425 ppm? Equally, other studies indicate different trials. It should be noted separately.

L493: after 21 days of feeding post-weaning.

Our responses to reviewers are in green. Line numbers we are referring to are those in the marked up file.

Reviewer #2 (Comments for the Author):

The manuscript provides valuable insights into the effects of zinc supplementation on the piglet gut microbiome. While the study shows promise, a major revision is required to improve clarity, strengthen the methodological rigor, and deepen the analysis. A more detailed explanation of the methodology, coupled with additional analyses, will enhance the manuscript and provide more compelling evidence regarding the effects of zinc supplementation on the microbiome of piglets.

1 There is a discrepancy in the reported "high zinc" concentrations. In the Abstract (line 22), it is stated as 2291 ppm, while in the Methods section (lines 108 and 458), it is listed as 2991 ppm. This inconsistency needs to be corrected.

Indeed, the right concentration was 2991 ppm. The text was modified accordingly (L22). We are thankful you noticed this typographical error.

2 The rationale behind the selection of zinc concentrations (low, moderate, high) is not adequately explained. The authors should clarify whether these concentrations reflect industry practices or are based on previous literature. Furthermore, it would be useful to reference regulatory standards to justify the high zinc levels used.

The rationale was introduced at L60-67, but to improve clarity, we included this sentence at L110-112 : ‘The low concentration meets the nutritional requirements of pigs, while the moderate and high concentrations reflect standard practice in industry.’

References to regulatory standards to justify the zinc levels used were provided at L78-82. While writing this manuscript, we even consulted with regulators at Health Canada, that ensured the information was up to date and complete.

L67: To add rigor, we removed one reference, since producers in China are using levels ≤ 500 ppm (Yang P, Wang HK, Li LX, Ma YX. 2021. The strategies for the supplementation of vitamins and trace minerals in pig production: surveying major producers in China. Anim Biosci 34:1350-1364.).

3 While pH and VFA are evaluated, additional physiological and immunological assessments (e.g., serum zinc levels, immune responses) should be included for a more comprehensive evaluation of the health impacts of zinc supplementation.

The pH and VFA of intestinal contents were included as they pertain to the gut microbiome, which was the focus of our study. Physiological assessments were included in a previous study to which we refer. Here are citations from this complementary work in reference #36 (Dalto DB, Audet I, Roy C, Novais AK, Deschene K, Goulet K, Matte JJ, Lapointe J. 2023. Effects of dietary zinc oxide levels on the metabolism of zinc and copper in weaned pigs. J Anim Sci 101):

‘ Serum, jejunum, liver, and kidney levels of Zn and Cu as well as mRNA abundance of genes related to Zn and Cu metabolism were analyzed. Zinc levels were greatest in 3,000Zn piglets from day 35 in all tissues ($P \leq 0.01$). In 3,000Zn piglets, mRNA expression of ZIP4 was reduced in jejunum whereas ZnT1 and MT3 were stimulated in jejunum and liver and MT1 in kidney ($P \leq 0.04$) from day 35. Copper levels were greatest in jejunum ($P = 0.06$) and kidney ($P \leq 0.01$; days 35 and 42 only) and lowest in liver and serum ($P \leq 0.01$) of 3,000Zn piglets.’

‘it was demonstrated that systemic Zn levels were not effectively regulated with supplementation levels at 3,000 mg of ZnO/kg of diet. In addition, this level of dietary ZnO increased the intestinal intracellular sequestration of Cu and impaired its renal reabsorption, negatively impacting hepatic, and systemic serum Cu concentrations. ’

4 The authors should provide justification for this timeframe or consider extending it to assess the long-term effects of zinc exposure on piglet growth, immunity, and microbiota. Including different growth stages would provide a better understanding of the long-term consequences.

We focused on the weaning period as it is a critical time for piglets, as described at L52-55, and it is the period where pharmacological levels of ZnO are used in the field. In fact, according to references #7-8, pharmacological levels of ZnO are usually limited to the first 14 days post-weaning, meaning that our study has already extended this period by seven days. Extending the experimental period further would not provide meaningful results in terms of field application.

5 In lines 192-194, the authors should describe their data preprocessing steps (e.g., quality control, contamination removal, normalization).

The data preprocessing steps were described at L187-190 and the link to the full details regarding the pipeline was provided (https://github.com/AAFC-Bioinfo-AAC/metagenomic_nf).

Multivariate analyses such as redundancy analysis (RDA) or PCA should be used to assess the impact of zinc on microbiota, and more sophisticated statistical models (e.g., structural equation

modeling) could help explore causal relationships. Data visualization could be enhanced with heatmaps, box plots, and violin plots, and p-value corrections (e.g., FDR, Bonferroni) should be applied to ensure the robustness of the results.

We calculated alpha indices presented as boxplots in Figure 3A and 3B which also included the results of an ANOVA. We also conducted NMDS, PERMANOVA and pairwise adonis analyses with the amplicon sequencing data (see Figure 3C and 3D), as well as discriminant analysis methods (see Figure 5).

The limited number of samples subjected to shotgun sequencing did not provide the required statistical power to conduct sophisticated statistical analyses.

6 detailed tables presenting microbial abundance data across different groups should be provided to enhance transparency and assist future replication of the study.

The microbial abundance data from amplicon sequencing is provided in a novel supplementary table: Table S3. It was introduced in the text at L298-299: ‘The abundances of taxa identify by amplicon sequencing are provided in Table S3.’

Reviewer #4 (Comments for the Author):

This study addresses an important and timely topic in swine nutrition and antimicrobial resistance management by investigating the effects of dietary zinc oxide supplementation on gut microbial community composition and antibiotic resistance genes in the ileum and colon. This research may have significant implications for improving antimicrobial stewardship in animal production systems while maintaining animal health.

However, I have identified several areas that require clarification and improvement before the manuscript can be considered for publication.

Specific comments: Abstract L29-30: Is this a positive or negative outcome? That context will be good in the abstract.

It is a negative outcome. ‘Of concern,’ was added to the sentence to stress that fact. (L28)

Keywords: Replace microbiome and resistome in the keyword. They already appear in title.

L48: The ‘Microbiome’ and ‘resistome’ keywords were replaced by ‘antimicrobial resistance’ and ‘metagenomics’.

Introduction L56-57: aside mortality, the significance of post weaning growth depression and morbidity is huge from the economic and animal welfare perspective. Re-phrase.

L57-58: We agree. This was added to the sentence: ‘animal welfare concerns and significant economic losses’

L64: 100 ppm of Zn per day? Per kg of feed intake? Specify.

L65: This information was added to increase clarity ‘100 mg of Zn per kg of feed.’

Ppm is a unit of concentration (ppm = mg/kg). The total amount of Zn ingested per day will vary according to feed intake. Therefore, we cannot add "per day" as the amount of feed ingested per day is variable between individuals (but not the concentration of Zn in the feed).

L105,229. Remove the initials from D.B Dalto et al. and F.H Wang

We agree that the initials may appear odd. However, this format is in line with the ASM Journals style for reference. Therefore, they were kept as is.

L115-117. The sentences here need re-written for clarity. Measurement of pH for colon and cecum; was this done on digesta sample collected before dilution? What quantity of digesta was sampled, with which equipment/device.

L119-120: The sentence was refined for clarity as such: ‘The pH was immediately measured directly into the cecum. Colon digesta (~ 5 g) was diluted 1:10 with tap water at pH 7, then the pH was measured. Ileum digesta samples (~ 2 g) were aliquoted and snap-frozen in liquid nitrogen.’

No specialized equipment was used for sampling; digesta were collected by manually expressing the intestinal tract.

L146: 0.3 g of 0.1 mm….?

L149: Despite the redundance, ‘Zirconia-Silicate beads’ was added to ensure clarity.

L428-430. Recast this sentence. Which of the ZnO levels decreased Lactobacillu57 or 2425 ppm? Equally, other studies indicate different trials. It should be noted separately.

L428-429: The sentence was revised to improve clarity: ‘A decrease in *Lactobacillus* was also observed in the high zinc treatment of a study comparing 57 ppm of ZnO with 2425 ppm (91), which was also reflected in other studies (92).’

L493: after 21 days of feeding post-weaning

L491: The text was modified to increase fluidity.

Re: Spectrum00059-26R1 (Characterization of the gut microbiome and resistome of piglets supplemented with low, moderate and high concentrations of zinc oxide)

Dear Dr. Dominic Poulin-Laprade:

Your manuscript has been accepted, and I am forwarding it to the ASM production staff for publication. Your paper will first be checked to make sure all elements meet the technical requirements. ASM staff will contact you if anything needs to be revised before copyediting and production can begin. Otherwise, you will be notified when your proofs are ready to be viewed.

Data Availability: ASM policy requires that data be available to the public upon online posting of the article, so please verify all links to sequence records, if present, and make sure that each number retrieves the full record of the data. If a new accession number is not linked or a link is broken, provide production staff with the correct URL for the record. If the accession numbers for new data are not publicly accessible before the expected online posting of the article, publication may be delayed; please contact ASM production staff immediately with the expected release date.

Publication Fees: For information on publication fees and which article types have charges, please visit our website. We have partnered with Copyright Clearance Center (CCC) to collect author charges. If fees apply to your paper, you will receive a message from no-reply@copyright.com with further instructions. For questions related to paying charges through RightsLink, please contact CCC at ASM_Support@copyright.com or toll free at +1-877-622-5543. CCC makes every attempt to respond to all emails within 24 hours.

ASM Membership: Corresponding authors may join or renew ASM membership to obtain discounts on publication fees. Need to upgrade your membership level? Please contact Customer Service at Service@asmusa.org.

Embargo Policy: A press release may be issued as soon as the manuscript is posted on the Spectrum Latest Articles webpage. The corresponding author will receive an email with the subject line "ASM Journals Author Services Notification" when the article is available online.

Cover Image Submissions: If you would like to submit a potential Cover Image, please email a file and a short legend to spectrum@asmusa.org. Please note that we can only consider images that (i) the authors created or own and (ii) have not been previously published. By submitting, you agree that the image can be used under the same terms as the published article. Image File requirements: TIF/EPS, 7.5 inches wide by 8.25 inches tall (at least 2,250 pixels wide by 2,475 pixels tall), minimum 300 dpi resolution (600 dpi preferred), RGB, and no figure elements, e.g., arrows or panel labels. The legend should be a short description of the image, 1-2 sentences recommended. Please use the ASM Journals Cover Image Sizing Tool (https://journals.asm.org/pb-assets/html/asm-cover-sizing-tool.html) to ensure that your proposed cover image meets our size requirements.

The ASM Journals program strives for constant improvement in our submission and publication process. Please tell us how we can improve your experience by taking this quick Author Survey.

Thank you for submitting your paper to Spectrum.

Sincerely,
Samara Mattiello
Editor
Microbiology Spectrum

Reviewer #2 (Comments for the Author):

The authors have addressed all of my comments and questions satisfactorily. I have no further concerns.
