# Supplementary material for: Characterization of the gut microbiome and resistome of piglets supplemented with low, moderate, and high concentrations of zinc oxide
Source: Microbiol Spectr. 2026 Sep 3;14(10):e00059-26. doi: 10.1128/spectrum.00059-26 (PMC13689069; doi:10.1128/spectrum.00059-26)
Supplement: Supplementary file 2 [file spectrum.00059-26-s0001.docx]

**Table S1.** Number of reads per metagenome after each quality filtering step.

| **Day** | **Treatment** | **Raw** | **Trimmed** | **Decontaminated** |
| --- | --- | --- | --- | --- |
| D21 | Low Zn | 72 439 806 | 71 483 36 | 69 986 569 |
|  | Moderate Zn | 67 376 715 | 66 490 026 | 65 729 543 |
|  | High Zn | 69 446 846 | 68 559 478 | 67 595 119 |
| D42 | Low Zn | 66 182 719 | 65 445 876 | 65 097 810 |
|  | Moderate Zn | 70 136 990 | 69 357 158 | 66 639 015 |
|  | High Zn | 86 305 473 | 83 981 602 | 83 900 242 |
